# COTI-2 induces cell apoptosis in pediatric acute lymphoblastic leukemia via upregulation of miR-203

**DOI:** 10.1080/21655979.2020.1729927

**Published:** 2020-02-17

**Authors:** Yingmeng Guo, Xia Zhu, Xuerong Sun

**Affiliations:** a Department of Pediatric Medicine, Linyi Central Hospital, Yishui, Shandong, China; b Department of Clinical Laboratory, Qingdao Women and Children’s Hospital, Qingdao, Shandong, China

**Keywords:** T-cell acute lymphoblastic leukemia, COTI-2, miR-203, caspase-3/9, apoptosis

## Abstract

COTI-2 is a third-generation thiosemicarbazone, which is effective against a diverse group of human cancer cell lines at nanomolar concentrations. COTI-2 also showed superior activity against tumor cells, in vitro and in vivo. As a high efficacy and low toxicity agent, it currently candidates in a phase I clinical study of gynecological malignancies and head and neck squamous cell carcinoma (HNSCC). However, its effect in pediatric T-cell acute lymphoblastic leukemia (T-ALL) is not clear. This study investigates the effect of COTI-2 on T-ALL Jurkat cells in vitro and in vivo. Jurkat cells were exposure to COTI-2 at different concentration and time. Cell apoptosis was detected by flow cytometry to examine the sensitivity of Jurkat cell lines treated with either COTI-2 alone or in combination with MiR-203 mimic or inhibitor in vitro. An orthotopic mouse model was used to examine the sensitivity of Jurkat cells treated with COTI-2 in vivo. Western blotting and RT-qPCR were performed to dissect molecular mechanisms. The results showed that COTI-2 promotes apoptosis of Jurkat cells in dose-and time-dependent way. Enforced expression of miR-203 promotes COTI-2-mediated cell apoptosis, whereas miR-203 silencing attenuates COTI-2-mediated cell apoptosis in Jurkat cells in vitro. COTI-2 is also effective against growth of Jurkat cells in vivo. Mechanistically, COTI-2 induced miR-203 upregulation and inhibited caspase-3/9 activaty leading to inhibition of cell apoptosis. Taken together, COTI-2 inhibits tumor growth in vitro and in vivo in Jurkat cells likely through miR-203-dependent mechanisms. COTI-2 may be a potential approach for T-ALL treatment.

## Introduction

Acute lymphoblastic leukemia (ALL) is a malignant transformation and proliferation of lymphoid progenitor cells in the bone marrow, blood and extramedullary sites. While 80% of ALL occurs in children, it represents a devastating disease when it occurs in adults [1]. The structure of treatment of adult ALL has been adapted from pediatric protocols. Unfortunately, while long-term survival approaches 90% for standard-risk pediatric ALL, the success rate is much more modest in adults [2]. Chemotherapy consists of induction, consolidation and long-term maintenance, with CNS prophylaxis given at intervals throughout therapy. However, due to high-risk disease characteristics and significant toxicity associated with chemotherapy in adults, outcomes are far less encouraging [3,4].

COTI-2, a novel small molecule currently in a phase I clinical study of gynecological malignancies and head and neck squamous cell carcinoma (HNSCC), was designed using CHEMSAS®, a proprietary computational platform.

It has recently reported that COTI-2 is effective against a diverse group of human cancer cell lines regardless of their tissue of origin or genetic makeup. Most treated cancer cell lines were sensitive to COTI-2 at nanomolar concentrations. When compared to traditional chemotherapy or targeted-therapy agents, COTI-2 showed superior activity against tumor cells, in vitro and in vivo [5]. Lindemann et al. reported that COTI-2 decreased clonogenic survival of HNSCC cells and potentiated response to cisplatin and/or radiation *in vitro* and *in vivo* through p53-dependent and p53-independent mechanisms [6]. In addition, combining COTI-2 with multiple chemotherapeutic and targeted agents enhances the activity of these drugs in vitro and in vivo, and no overt toxicity was observed in the combination treatment groups in vivo. Furthermore, some chemo-resistant tumor cell lines only showed mild cross-resistance to COTI-2 while most remained sensitive to it [7]. However, the mechanism of action of COTI-2 is still unclear.

MicroRNA (miRNA) is a single-stranded, non-coding RNA molecule of 22–25 nucleotides, which leads to down-regulation of target protein expression [8]. Recently, hypermethylation of *miR-203* has been reported in chronic myeloid leukemia (CML) and acute myeloid leukemia (AML), conferring proliferative advantage in tumor cells [9,10].

In this study, we aimed to study the anti-cancer role and potential mechanism of COTI-2 in acute lymphoblastic leukemia (ALL) cells *in vitro* and *in vivo*. Our results show that COTI-2 effectively induced apoptosis and inhibited growth of leukemia cells via activating miR-203.

## Materials and methods

### Cytotoxic drugs

COTI-2 was supplied by Critical Outcome Technologies Inc. (London, Ontario). COTI-2 was dissolved in 100% dimethyl sulfoxide stock solution and diluted in medium plus FBS such that final DMSO concentrations were 0.5–1.0% depending on the experiment

### Cell culture

Jurkat cells were purchased from AmericanType Culture Collection (ATCC, Manassas, VA, USA) and cultured in RPMI 1640 supplemented with 10% heat inactivated fetal calf serum, 100 U/ml penicillin, 100 μg/ml streptomycin and 2 mM L-glutamine (Life Technologies, Carlsbad, CA).

### Transfection of cells with the miR‐203 mimics vector or the inhibitors vector

For miR‐203 overexpression, the miR‐203 mimic or corresponding negative control (miR‐NC) was purchased from GenePharma (Shanghai, China). Jurkat cells were transfected with either the miR‐203 mimic or miR‐NC at a final concentration of 100 nM using Lipofectamine® 2000 (Invitrogen) according to the manufacturer’s protocol. Cells were used for miR‐203 expression analysis or other experiments after 48 h of transfection. For miR‐203 inhibition, Jurkat cells were treated with final concentration of 100 nM miR‐203 inhibitor (Invitrogen) for 48 h, then the effect of miR‐203 on related gene expression was detected.

### qPCR for miR-203

Total RNA from tissue and cells were isolated using TRIzol reagent (Invitrogen) to obtain miRs according to the manufacturer’s instructions. All RNA extractions were carried out in a sterile laminar flow hood using RNase/DNase-free laboratory ware. The single-tube TaqMan miRNA assay (Applied Biosystems, CA, US) was used to detect and quantify mature miR-203, using ViiA7 RT reader (Applied Biosystems, CA, US); the protocol was performed for 38 cycles at 95°C for 3 min, 95°C for 15 s, and 60°C for 30 s. miR-203 expression was normalized on U6, and then expressed as fold change (2^ΔΔCt^).

### Western blotting analysis

Cells were seeded at a density of 3 × 10^5^ per well in 6-well plates and allowed to attach for 24 h. After various treatments, the whole cell lysates were subjected to Western blotting analysis. Total proteins were extracted by using RIPA lysis buffer containing 10 mm Tris-HCl (pH 7.4), 150 mm NaCl, 1 mm EDTA, 1% Triton X-100 (w/v), 0.5% sodium deoxycholate, and 0.1% SDS with protease and phosphatase inhibitor mixture. The equal amounts of protein were separated in 10% SDS-PAGE and transferred to a nitrocellulose membrane. The membranes were incubated with primary antibodies overnight at 4°C. The following primary antibodies were used: PARP, cleaved-caspase-3 and cleaved-caspase-9. The corresponding HRP-conjugated secondary IgG antibodies were used, and immunoreacted proteins were detected with chemiluminescence reagent.

### Apoptosis assay

Apoptotic cells were measured by staining with FITC-conjugated Annexin V/propidium iodide (PI) (BD Pharmingen, San Diego, CA, USA) and determining with ﬂow cytometer according to the manufacturer’s instructions. Both early apoptotic (Annexin V-positive, PI-negative) and late apoptotic (Annexin V-positive and PI-positive) cells were included in cell death determinations.

### Xenograft model and miR-203 evaluation

Nude mice (5 weeks old) were purchased from Vital River Laboratories (Beijing, China). Animal experiments were approved by the Institutional Animal Care and Use Committee (IACUC) of the university. Jurkat cells (5 x 10^6^/0.2 ml per mouse) were suspended in sterile PBS and injected subcutaneously into the right ﬂank of the mice (n = 10 mice per treatment group). When tumors reached a palpable volume of approximately 75–100 mm^3^ (3–4 weeks), treatment with COTI-2 (10 mg/kg, 5 days a week for 7 weeks) or saline intraperitoneal injection (IP) began. Tumor growth was measured every 4 days by caliper measurement. Tumor volumes were determined by a caliper and calculated according to the formula (width2 x length)/2. Absolute quantities of miR-203 in tumor tissue were obtained by qRT-PCR .

### Immunohistochemical evaluation

Tumor tissue sections (4μm) were subjected to immunohistochemical staining (IHC) for cleaved-caspase-3. The percentage of positively-stained area with 40 fields of view was analyzed by Image-pro plus 6.0 (Media cybernetics).

### TUNEL evaluation

Apoptosis in tumor tissue sections was determined using In Situ Cell Death Detection kit (Roche, Mannheim, Germany). Brieﬂy, tumor tissue sections of formalin-ﬁxed, parafﬁn-embedded specimens were dewaxed in xylene

and rehydrated in a graded series of ethanol. The tumor samples were incubated with proteinase K (2 mg/ml), and the TUNEL staining was performed according to the manufacturer’s instructions.

### Densitometry and statistical analysis

Statistical analysis was performed using SPSS.22 Software. One-way analysis of variance (ANOVA) test was used for comparisons when more than two groups were analyzed and Student’s *t*-test when two groups were analyzed. ANOVA was performed using the Bonferroni *post-hoc* test. Values of *P* < 0.05 were considered significant.

## Results

### COTI-2 induces apoptosis in dose- and time-dependent manners

**Figure 1.**
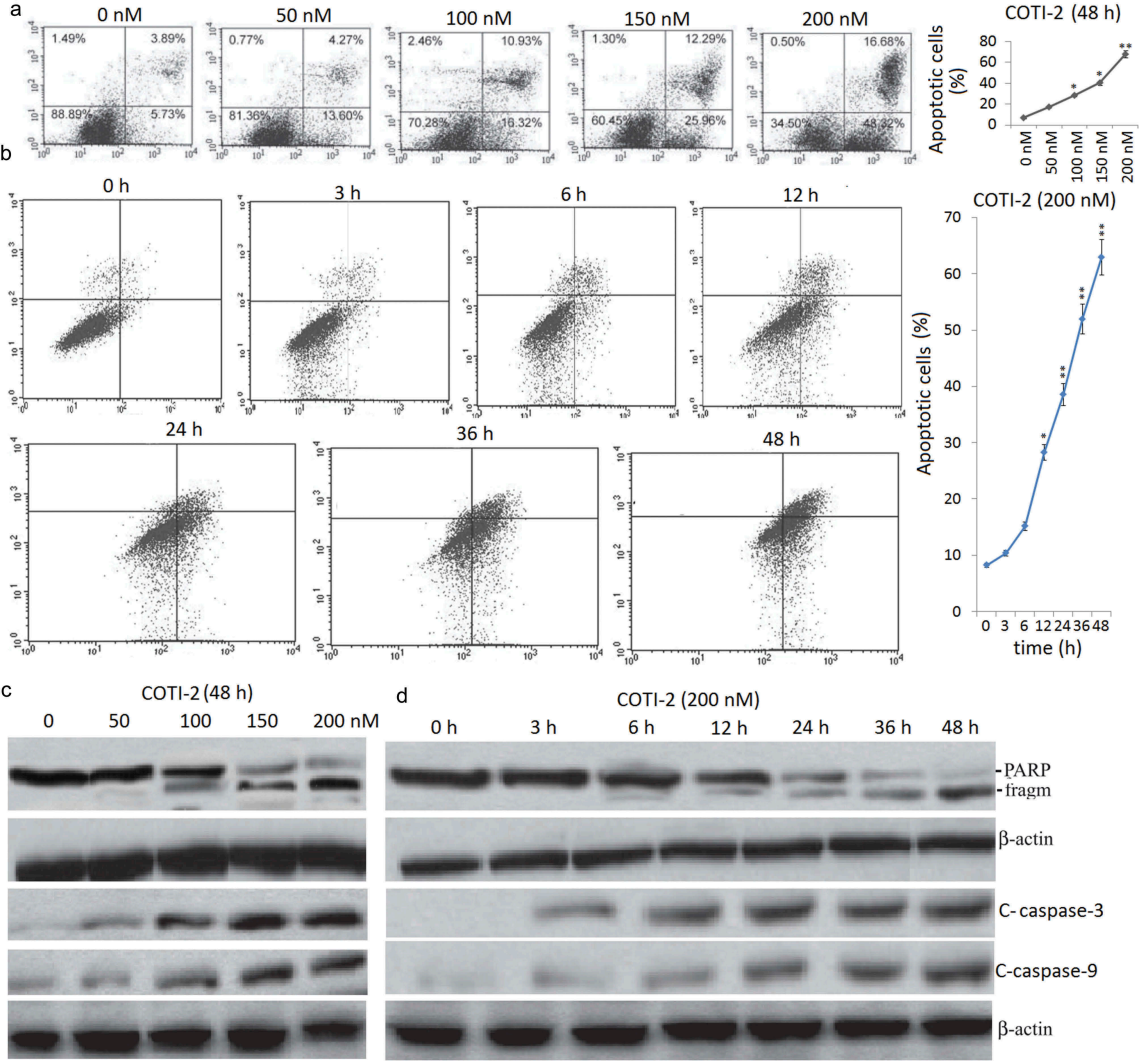
Treatment with COTI-2 results in caspase activation and apoptosis in dose- and in time-dependent manners in Jurkat cells. (a), Jurkat cells were treated with 0, 50, 100, 150, and 200 nM **COTI-2** for 48 h. (b) The cells were treated with 200 nM BITC for 0–48 h. In (a) and (b), cells were stained with Annexin V/PI, and the percentage of apoptotic cells was determined using ﬂow cytometry. (c,d), After treatment with **COTI-2**, total cellular extracts, nuclear extracts, and cytosolic fractions were prepared and subjected to western blot analysis using antibodies against PARP, cleaved-caspase (C-Caspase)-9, cleaved-caspase-3. Each lane was loaded with 30 mg of protein. Two additional studies yielded equivalent results.

A dose-dependent study in Jurkat cells revealed a moderate increase in apoptosis 48 h after exposure to 100 nM COTI-2 and very extensive apoptosis at concentrations of 200 nM (Figure 1(a)). Time-course analysis of cells exposed to 200 nM COTI-2 demonstrated a signiﬁcant increase in apoptosis as early as 6 h. These events became apparent after 24 h of drug exposure, and reached maximal levels after 48 h (Figure 1(b)). Consistent with these ﬁndings, the same COTI-2 concentrations and exposure intervals resulted in cleavage/activation of caspase-9 and −3, and degradation of poly-ADP-ribose polymerase (PARP) (Figure 1(c,d)).

### Exposure of jurkat cells to COTI-2 results in the upregulation of miR-203

**Figure 2.**
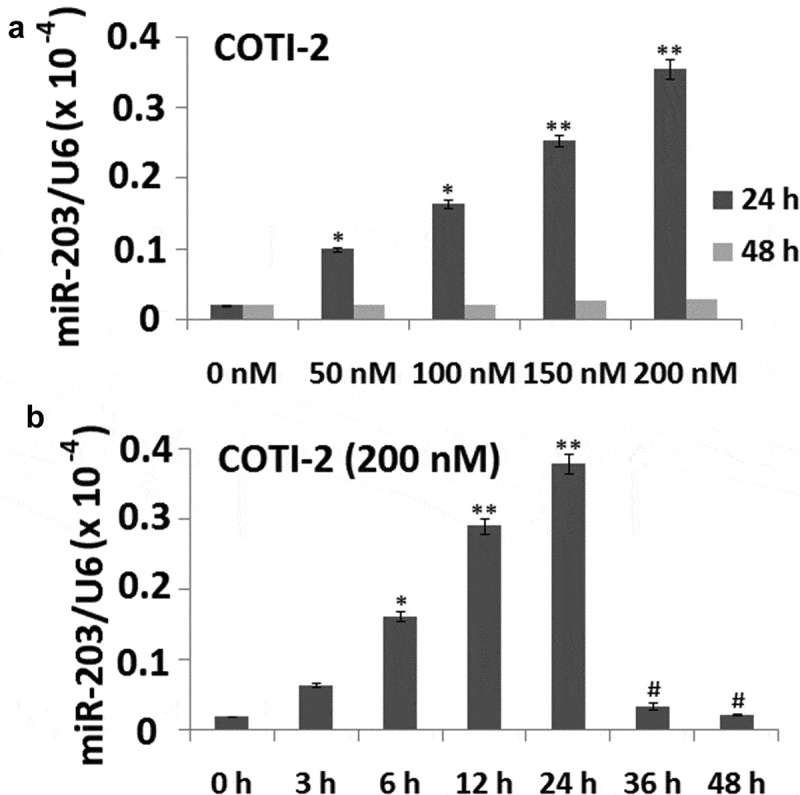
Treatment with COTI-2 results in increased miR-203 expression in dose- and in time-dependent manners in Jurkat cells. (a), Jurkat cells were treated with 0, 50, 100, 150, and 200 nM **COTI-2** for 48 h. (b) The cells were treated with 200 nM BITC for 0–48 h. In (a) and (b), miR-203 expression was detected by qRT-PCR assay.

The effects of COTI-2 on the expression of miR-203 was examined in Jurkat cells. A marked dose-dependent increase of miR-203 expression was noted in COTI-2-treated cells in 24 h (Figure 2(a)). At 48 h, the miR-203 levels reached the normal level (Figure 2(a)).

Exposure of cells to 200 nM COTI-2 for 6 h resulted in a modest increase in levels of miR-203. These events became apparent after 12 h. Then miR-203 expression was increased and reached the highest level after 24 h of drug exposure, and reached the normal level at 48 h (Figure 2(b)).

### Enforced expression of miR-203 enhances coti-2-mediated apoptosis

**Figure 3.**
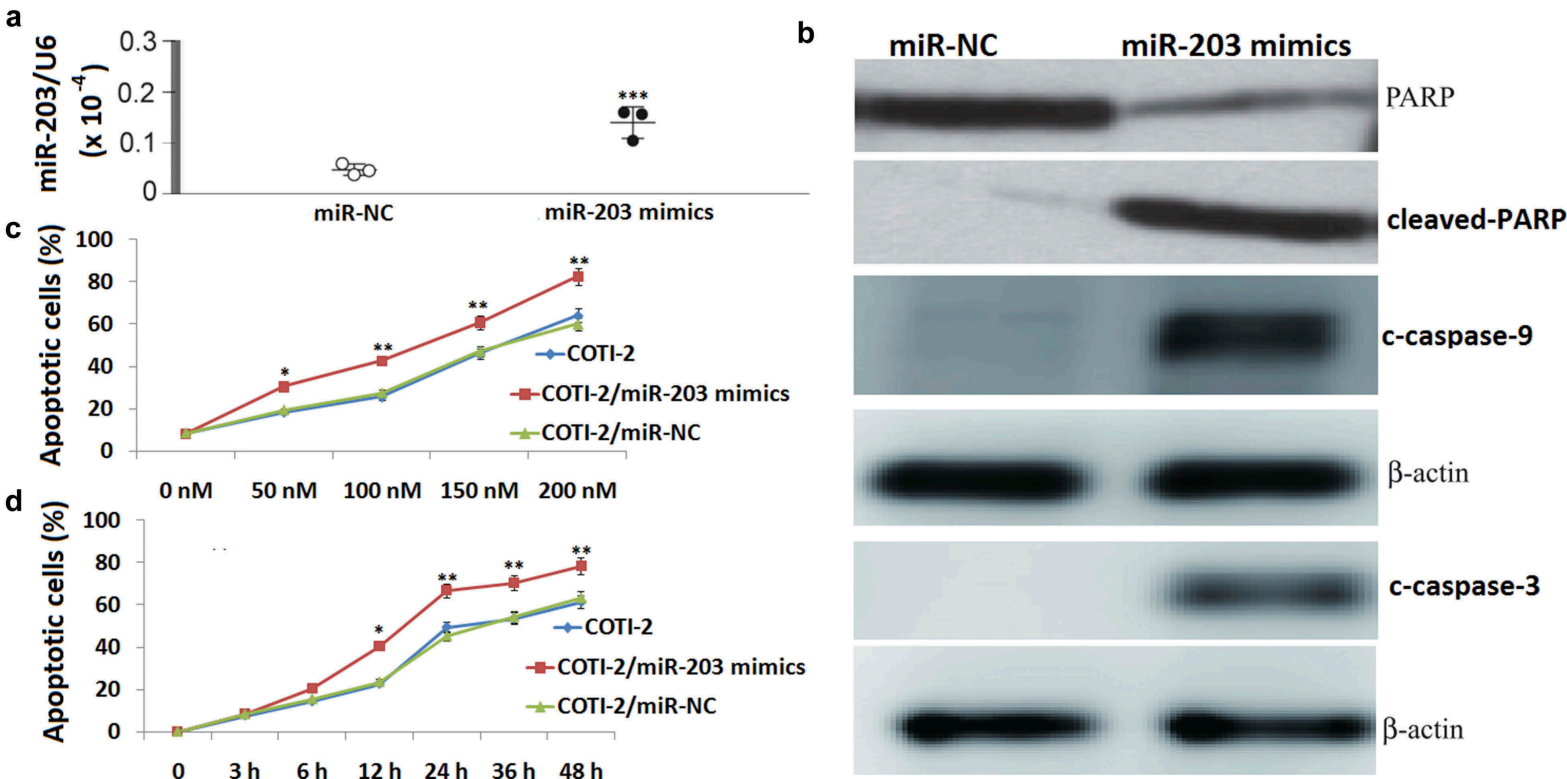
Enforced expression of miR-203 enhanced BITC-mediated apoptosis in Jurkat cells. Total cellular extracts were prepared from Jurkat cells transfected with miR-NC (control) and miR-203 mimic, and then subjected to qRT-PCR assay for miR-203 (a), and western blot assay for cleaved caspase-9 and −3 and PARP (b). (c), Jurkat cells transfected with miR-NC or miR-203 mimic were treated with 0, 50, 100, 150, and 200 nM **COTI-2** for 48 h. After treatment, cells were stained with Annexin V/PI, and apoptosis was determined using ﬂow cytometry. (d), Jurkat cells transfected with miR-NC or miR-203 mimic were treated with 200 nM **COTI-2** for 3–48 h. After treatment, cells were stained with Annexin V/PI, and apoptosis was determined using ﬂow cytometry. Significant difference from controls, Student’s *t*-test, *p < 0.05; **p < 0.01;***P < 0.001.

To further conﬁrm the functional role of miR-203 in COTI-2-mediated lethality in leukemia cells, Jurkat cells expressing miR-203 mimics were employed. Exposure of Jurkat cells to miR-203 mimics resulted in a signiﬁcant increase of miR-203 expression compared with miR-NC transfected Jurkat cells (Figure 3(a)). In addition, miR-203 overexpression promotes the activation of caspase-9 and −3 and degradation of PARP (Figure 3(b)). Furthermore, miR-203 overexpression in Jurkat cells increased COTI-2-mediated apoptosis compared with control cells in a dose- and time- dependent manner (Figure 3(c,d)). Taken together, these ﬁndings indicate that miR-203 upregulation has a signiﬁcant functional role in COTI-2-mediated lethality.

### Knockdown of miR-203 substantially attenuates COTI-2-mediated apoptosis

**Figure 4.**
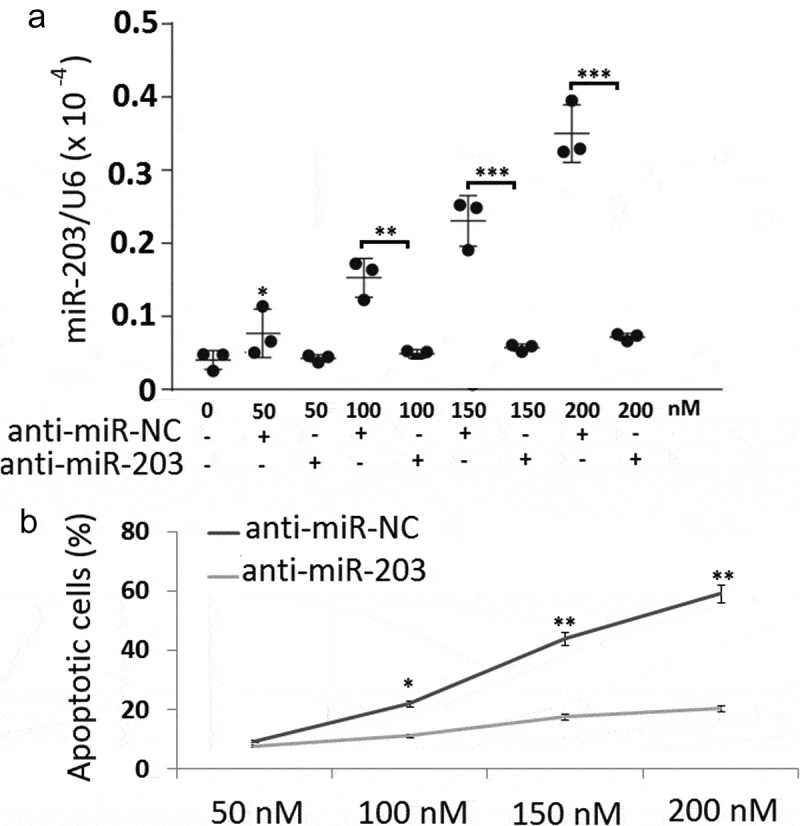
Downregulation of miR-203 inhibited BITC-mediated apoptosis in Jurkat cells. (a), Total cellular extracts were prepared from Jurkat cells transfected with NC inhibitor (control) and miR-203 inhibitor, and then subjected to qRT-PCR assay for miR-203; (b), Jurkat cells transfected with miR-NC or miR-203 mimic were treated with 0, 50, 100, 150, and 200 nM **COTI-2** for 48 h. After treatment, cells were stained with Annexin V/PI, and apoptosis was determined using ﬂow cytometry. Significant difference from controls, Student’s *t*-test, *p < 0.05; **p < 0.01;***P < 0.001.

To further conﬁrm the functional role of miR-203 in COTI-2-mediated lethality in leukemia cells, Jurkat cells expressing miR-203 inhibitors were employed. The results showed that miR-203 inhibitor transfection significantly decreased COTI-2-induced miR-203 expression in Jurkat cells (Figure 4(a)). Signiﬁcantly, miR-203 downregulation attenuated COTI-2-mediated apoptosis (Figure 4(b)), and completely inhibited the activation of caspase-9 and −3 and degradation of PARP. These data indicate that miR-203 downgulation has a signiﬁcant functional role in COTI-2-mediated apoptosis.

### COTI-2 exhibits antitumor activity in xenografts of leukemia Jurkat cells by induction of apoptosis and upregulation of miR-203

**Figure 5.**
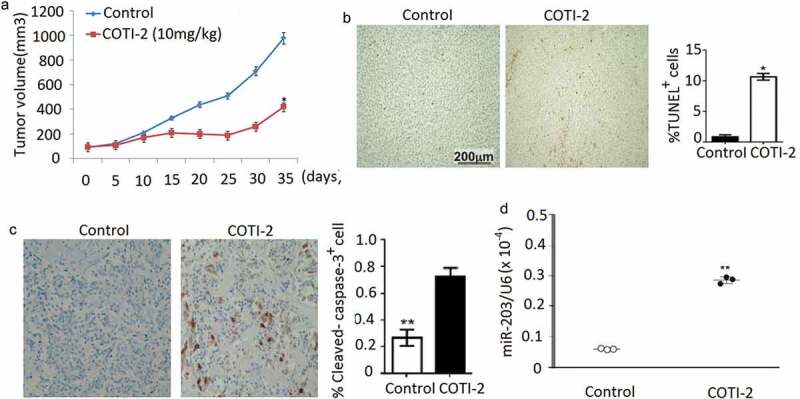
COTI-2 treatment inhibits Jurkat xenograft growth (a). Jurkat cells (5 × 10^6^) were injected into the flanks of mice (n = 5 mice per group). Xenografts were allowed to reach 75 ~ 100 mm^3^ before IP treatment initiation with COTI-2 (10 mg/kg, 5 days a week for 7 weeks) or saline alone. Tumor growth was measured every 5 days by caliper measurement. (b), Tumors were obtained from animals 35 days after drug exposure. Tumors were ﬁxed and stained to examine apopptosis using TUNEL assay. (c), The levels of cleaved-caspase-3 was detected by immunohistochemistry; (d), The levels of miR-203 was detected by qRT-PCR. Significant difference from controls, Student’s *t*-test, *p < 0.05. **p < 0.01.

We assessed the effectiveness of COTI-2 in inhibiting the growth of Jurkat xenografts in immunocompromised mice when administered intraperitoneally (IP). COTI-2 significantly inhibited tumor growth in the Jurkat xenografts at a dose of 10 mg/kg (Figure 5(a)). We further determined apoptosis in tumor tissue of leukemia xenograft using TdT-mediated dUTP-biotin nick-end labeling (TUNEL) assay. TUNEL-positive apoptotic cells of tumor sections signiﬁcantly increased in COTI-2-treated Jurkat xenograft mice compared with the control groups (Figure 5(b)). Exposure of mice to COTI-2 also caused a rapid increase in immunoreactivity for cleaved-caspase-3 in tumor sections (Figure 5(c)), indicative of apoptosis. Furthermore, miR-203 expression in tumor sections of Jurkat xenograft mice increased upon COTI-2 treatment (Figure 5(d)). Such ﬁndings suggest that COTI-2-mediated antileukemic activity *in vivo* is associated with the upregulation of miR-203.

## Discussion

The results of this study indicate that treatment with COTI-2 results in apoptosis in human leukemia cells. Notably, this study demonstrates for the ﬁrst time that upregulation of miR-203 has an important role in COTI-2-mediated lethality.

COTI-2 appears to be a cytotoxic agent since it inhibited cellular proliferation by inducing apoptosis in cancer cells [11]. In the present study, COTI-2 demonstrated effective anti-proliferative and pro-apoptotic activity against leukemia cell lines in vitro.

Extensive evidence is accumulating that miR-203 expression has a critical role in inducing cell apoptosis and inhibiting the survival of transformed cells [12,13], particularly those of hematopoietic origin [9]. The development of anticancer agents that enhanced miR-203 levels has been the focus of intense interest. Indeed, a number of studies have documented miR-203 upregulation during apoptosis by a variety of agents, including lncRNA MALAT1 [14], Sevoflurane [15], Berberine, the active compound of traditional Chinese medicine Huang Lian [16]. Evidence revealed that miR-203 levels are regulated through several different mechanisms [17–19].

In the present study, enforced expression of miR-203 markedly enhanced COTI-2-mediated lethality in Jurkat cells, arguing that upregulation of miR-203 has a critical role in COTI-2-related lethality. Consistent with this notion, targeting miR-203 largely inhibited caspase activation. These ﬁndings are consistent with previous study, which demonstrated that miR-203 operates upstream of caspases signals [20]. In addition, the miR-203 overexpression in Jurkat cells led to a signiﬁcant increase in COTI-2-mediated caspase activation and apoptosis. Such ﬁndings are accordant with the studies that indicated that upregulation of miR-203 through miR-203 mimic transfection is sufﬁcient to induce apoptosis in multiple myeloma cells [21].

Previous studies have shown that COTI-2 markedly inhibits tumor growth of human HNSCC cell xenografts [6]. However, little is known about inhibitory effects of COTI-2 on tumor growth of human leukemia xenograft model.

In our studies using a nude mice Jurkat xenograft model, tumor volumes were reduced compared with controls after COTI-2 treatment, indicating an antileukemia activity of this compound. To further validate the apoptotic mechanism found in vitro, we next examined the TUNEL staining, cleaved-caspase-3, and miR-203 expression in tumor specimens obtained from control- and COTI-2-treated animals. The increase in TUNEL-positive cells, caspase-3 activation, and the increase in miR-203 expression were detected in the COTI-2-treated xenografts compared with the control group. To the best of our knowledge, this is the ﬁrst report that describes an effective extrapolation of the in vitro apoptosis-inducing effects of COTI-2 on the leukemia cells to the in vivo situation.

## Conclusion

In conclusion, these ﬁndings indicate that COTI-2 effectively induces apoptosis in human leukemia cell lines and leukemia xenograft. This effect occurs in association with the upregulation of miR-203, leading to caspase activation. This study could provide a better understanding of how COTI-2 exerts its antileukemic activity in vivo and aid in developing this compound to treat leukemia and possibly other hematologic malignancies.
